# Genomic epidemiology of extended-spectrum beta-lactamase-producing *Escherichia coli* and *Klebsiella pneumoniae* in Mwanza, Tanzania

**DOI:** 10.1016/j.jgar.2026.01.010

**Published:** 2026-03

**Authors:** Vitus Silago, Benson R. Kidenya, Katarina Oravcova, Louise Matthews, Conjester I. Mtemisika, Stephen E. Mshana, Heike Claus, Jeremiah Seni

**Affiliations:** a Department of Microbiology and Immunology, Weill Bugando School of Medicine, Catholic University of Health and Allied Sciences, Mwanza, Tanzania; b Institute for Hygiene and Microbiology, University of Würzburg, Würzburg, Germany; c Department of Biochemistry and Molecular Biology, Weill Bugando School of Medicine, Catholic University of Health and Allied Sciences, Mwanza, Tanzania; d School of Biodiversity, One Health and Veterinary Medicine, University of Glasgow, Glasgow, UK; e Department of Molecular Biology, Clinical Laboratory, Bugando Medical Centre, Mwanza, Tanzania

**Keywords:** Bacterial genomic evolution, Clonal diversity, *E. coli*, Extended-spectrum beta-lactamase, *Klebsiella pneumoniae*, Whole genome sequencing

## Abstract

**Background:**

Extended-spectrum beta-lactamase-producing *Escherichia coli* (ESBL-EC) and *Klebsiella pneumoniae* (ESBL-KP) represent major clinical threats globally. Genomic epidemiological data remain scarce in low- and middle-income countries, limiting a comprehensive understanding of antimicrobial-resistant pathogen diversity and clonal distribution. The present study investigated the genomic epidemiology of ESBL-EC and ESBL-KP isolates in Mwanza, Tanzania.

**Methods:**

This cross-sectional hospital-based study employed whole-genome sequencing to characterise ESBL-EC (*n* = 39) and ESBL-KP (*n* = 49) isolated from patients with bloodstream, urinary tract, and wound infections at a zonal referral hospital between June 2019–June 2020 and March–August 2023.

**Results:**

Thirteen sequence types (STs) were identified among ESBL-EC, predominantly ST131 (30.7%) and ST648 (28.2%). ESBL-KP comprised 15 STs, with ST2390 (24.5%) and ST17 (18.4%) being the most common. The *bla*_CTX−M-15_ gene was detected in 87.2% of ESBL-EC and 95.9% of ESBL-KP. IncFII was the dominant plasmid replicon in ESBL-EC (63.9%) and ESBL-KP (83.7%), while repB was detected exclusively in ESBL-KP (28.6%), particularly among ST2390. ESBL-EC showed significantly higher resistance to ciprofloxacin (*P* < 0.01), whereas ESBL-KP demonstrated higher resistance to gentamicin and piperacillin-tazobactam (both *P* < 0.01). The cgMLST-based Neighbour-Joining phylogenetic analysis revealed substantial genetic diversity and identified clonal clusters involving the high-risk clone ESBL-EC ST131. Clusters of ESBL-EC ST131 and ST648 were observed across medical and neonatology wards, while ESBL-KP ST2390 clusters were mainly confined to neonatology wards.

**Conclusion:**

This study highlights clonal clusters, the first report of ESBL-KP ST2390, and the predominance of the virulent high-risk clone ESBL-EC ST131 in Mwanza, Tanzania. Underscoring the critical need for reinforced infection control strategies and genomic surveillance.

## HIGHLIGHTS

• Predominance of globally recognised high-risk clone extended-spectrum beta-lactamase (ESBL)-producing *Escherichia coli* ST131.
• First documentation and predominance of ESBL-producing *Klebsiella pneumoniae* ST2390 associated with bloodstream infections in the neonatology units.
• The cgMLST-based Neighbour-Joining (NJ) phylogenetic analysis revealed clonal clusters involving high-risk clone ESBL-EC ST131 in medical and neonatology wards, and newly detected ESBL-KP ST2390 in the neonatology ward/unit.

## 1 Introduction

Antimicrobial resistance (AMR) represents a critical global health threat, with extended-spectrum beta-lactamase (ESBL)-producing Enterobacterales (PE), particularly *Escherichia coli* and *Klebsiella pneumoniae*, at the forefront [1]. These pathogens cause life-threatening bloodstream infections (BSIs), urinary tract infections (UTIs), and skin and soft tissue infections (SSTIs), with disproportionate high burden in low- and middle-income countries (LMICs) where diagnostic capacity and antimicrobial stewardship programs are limited [2,3].

A global burden study from 2019 estimated nearly 929 000 deaths associated with drug-resistant *E. coli* and *K. pneumoniae*, with significantly higher mortality in sub-Saharan African countries [2]. Globally, high-risk clones of ESBL-producing *E. coli* (ESBL-EC), i.e., sequence type 131 (ST131) and serotype O25:H4 and ESBL-producing *K. pneumoniae* (ESBL-KP), i.e., ST307 and serotype O2afg:KL102 have emerged as key resistance drivers [4,5]. In Tanzania, limited research-based genomic data are showing predominance of ESBL-EC ST131 and ESBL-KP ST14 and ST45 implicated in both gastrointestinal colonisation and infections [[6], [7], [8]]. These STs exhibit multidrug resistance, often harbouring *bla*_CTX−M-15_ alongside resistance to fluoroquinolones and aminoglycosides, with enhanced pathogenicity through virulence factors related to adhesion, invasion, immune evasion, and iron acquisition [4,5].

The World Health Organization (WHO) has prioritised third-generation cephalosporin-resistant Enterobacterales comprising ESBL-EC and ESBL-KP in its Global Action Plan on AMR and its Bacterial Priority Pathogens Lists, 2017 and 2024 [[9], [10], [11]]. Despite adopting national action plans on AMR (NAP-AMR) across LMICs, including Tanzania [12], genomic AMR surveillance data shortages persist, hampering infection prevention and control (IPC) efforts. Moreover, understanding local clonal clusters and resistance mechanisms is essential for effective targeted public health interventions. This study bridges this knowledge gap by utilising whole genome sequencing (WGS) to characterise ESBL-EC and ESBL-KP isolates causing BSIs, UTIs, and SSTIs among patients admitted or attending at Bugando Medical Centre (BMC), a zonal referral hospital in Mwanza, Tanzania. This research seeks to generate insights to guide targeted surveillance and IPC strategies in resource-limited healthcare settings through a comprehensive analysis of genomic epidemiology (diversity, resistance determinants, virulence factors, and clonal relationships) of ESBL-PE.

## 2 Materials and methods

### 2.1 Study design

This cross-sectional hospital-based study employed WGS to characterise ESBL-EC and ESBL-KP isolates recovered from blood, urine, and pus samples of patients with clinical diagnoses of BSIs, UTIs, and/or SSTIs, respectively. The samples were collected from patients attending or admitted to BMC, a zonal referral hospital in Mwanza, Tanzania, during June 2019–June 2020 and March–July 2023, as part of a previous research project [13,14].

### 2.2 Bacterial isolation and antimicrobial susceptibility testing

Routine bacterial culture, biochemical tests, and disk diffusion methods by Kirby-Bauer technique were employed for the isolation, species identification, and antimicrobial susceptibility testing (AST) of bacterial pathogens implicated in BSIs, UTIs, and SSTIs following standard operating procedures [15,16]. All isolates were stored at −80 °C until further analysis. Species verification was performed using an automated analyser, VITEK MS (Matrix-assisted laser desorption/ionisation – time of fright [MALDI-TOF]; bioMérieux, Germany). Whereas AST was performed using the VITEK 2 (bioMérieux, Germany) system with the AST-N214 card to determine the minimum inhibitory concentrations (MICs) of bacterial pathogens against antimicrobial agents. The AST-N214 card included ampicillin, cefpodoxime, cefuroxime, cefotaxime, trimethoprim-sulfamethoxazole, gentamicin, ciprofloxacin, piperacillin-tazobactam, tigecycline, and meropenem. The AST also included screening for ESBL production and assessment of multidrug resistance levels. Interpretation of MIC values was performed using the EUCAST 2023 guidelines [17].

### 2.3 Isolate selection and DNA extraction for WGS

ESBL-positive *E. coli* and *K. pneumoniae* strains classified as multidrug-resistant Gram-negative bacteria (MRGN) level ≥3 (resistance to at least one or more antimicrobials from three or more different antimicrobial classes) were selected for WGS. The isolates were subcultured on 5% sheep blood agar and incubated aerobically at 35 ± 2 °C for 24 h prior to DNA extraction using the Wizard® Genomic DNA Purification Kit (Promega, Germany). DNA samples with absorbance ratio A260/A280 ≥1.80 and A260/A230 2.0–2.2 by Nanodrop Spectrophotometer (bioMérieux, Germany) were accepted for WGS and stored at −20 °C.

### 2.4 WGS and bioinformatics analysis

WGS was performed on an Illumina NextSeq 500/550 instrument (Illumina, San Diego, CA) using Nextera XT library preparation and 2 × 150 bp paired-end sequencing. Raw sequences underwent quality control using FastQC, trimming with Trimmomatic, and *de novo* assembly using Velvet in Ridom SeqSphere+ *v*.8.4.0 (Ridom GmbH, Münster, Germany; https://www.ridom.de/seqsphere/). MLST sequence types were identified using the PubMLST database (https://pubmlst.org/mlst) within Ridom SeqSphere+. Additionally, Neighbour Joining (NJ) phylogenetic trees for ESBL-EC and ESBL-KP were later generated using the Bruker MBioSEQ Ridom Typer (formerly Ridom SeqSphere+), with the clonal clustering threshold set at ≤10 cgMLST allelic distance. In the current study, a clonal cluster represents bacterial isolates with minimal genetic divergence (≤10 cgMLST allelic differences) within a clone. A clone denotes a set of bacterial isolates forming a genetically uniform group as a result of recent shared ancestry (e.g., ST131), whereas a lineage represents a higher-order phylogenetic group encompassing multiple related clones with shared ancestry.

Comprehensive genomic characterisation employed multiple bioinformatics tools from the Center for Genomic Epidemiology (CGE) of the Technical University of Denmark (https://www.genomicepidemiology.org/services/): SeroTypeFinder 2.0 [18] for serotype identification, PlasmidFinder 2.1 [19,20] for plasmid replicon detection and typing, and ResFinder 4.6.0 [19,21] for antimicrobial resistance gene (ARG) identification. When ResFinder failed to detect third-generation cephalosporin resistance genes, the Resistance Gene Identifier (RGI) v6.0.5 [22] from the Comprehensive Antibiotic Resistance Database (CARD v4.0.1; https://card.mcmaster.ca/analyze/rgi) was utilised. All analyses performed using bioinformatics tools within CGE and CARD were conducted with default settings.

The CGE VirulenceFinder 2.0 [19] was used for virulence gene detection in ESBL-EC, whereas the virulence factor database (VFDB; https://www.mgc.ac.cn/cgi-bin/VFs/v5/main.cgi) and Pathogenwatch (https://pathogen.watch/) were used to determine virulence factors and serotypes in ESBL-KP, respectively. Additionally, Kleborate [23] on Galaxy Europe (https://usegalaxy.eu/) was used for species identification and virulence scoring in ESBL-KP. Moreover, the ClermonTyping [24,25] was used for phylotyping of ESBL-EC into major phylogroups.

### 2.5 Statistical analysis

Data were analysed using STATA 15.0 [26]. Chi-square or Fisher's exact tests were used to compare categorical variables, while Wilcoxon rank-sum tests compared continuous variables. Statistical significance was set at *P* ≤ 0.05 and 95% confidence interval.

## 3 Results

### 3.1 Characteristics of sequenced isolates

A total of 88 ESBL-producing *E. coli* and *K. pneumoniae* isolates classified as MRGN level ≥3 were selected for WGS. These isolates were obtained from patients with a median age of 9.5 y [IQR: 0–42.5], the majority of whom were inpatients (96.6%; *n* = 85). Most isolates originated from blood samples (43.2%; *n* = 38), primarily from neonates admitted to the neonatology unit (34.1%; *n* = 30). Furthermore, 30.7% (*n* = 27) of patients reported a history of fever, 19.3% (*n* = 17) had used antibiotics within the past 3 months, and 13.6% (*n* = 12) had comorbid conditions (Table 1).

**Table 1:** The demographic and clinical characteristics of patients whose ESBL-EC and ESBL-KP were sequenced.

Variables		Frequency	Percentage
		Median [IQR]/*n*	%
Median [IQR] age in years		9.5 [0–42.5]	–
Sex	Female	45	51.1
	Male	43	48.9
Residence	Rural	45	51.1
	Urban	43	48.9
Patient category	Inpatient	85	96.6
	Outpatient	3	3.4
Ward	Neonatology	30	34.1
	Surgical	25	28.4
	Medical	23	26.1
	Others	10	11.4
Sample type	Blood	38	43.2
	Pus	33	37.5
	Urine	17	19.3
History of fever	No	61	69.3
	Yes	27	30.7
History of antibiotic use	No	71	80.7
	Yes	17	19.3
History of admission	No	83	94.3
	Yes	5	5.7
Comorbidities*	No	76	86.4
	Yes	12	13.6

⁎ Comorbidities: Hypertension only (*n* = 4), sickle cell disease (*n* = 3), diabetes mellitus only (*n* = 2), hypertension and diabetes mellitus (*n* = 2), and HIV (*n* = 1).

### 3.2 Sequence types, serotypes, and plasmid replicons

Of the 88 sequenced ESBL-PE isolates, 39 were ESBL-EC and 49 were ESBL-KP. Among the ESBL-EC, 13 sequence types (STs) were identified, with ST131 (30.7%; *n* = 12) and ST648 (28.2%; *n* = 11) being the most prevalent. The majority of ESBL-EC ST131 (7 out of 12) were isolated from urine samples. Nearly all ESBL-EC (94.9%; *n* = 37) were serotypable, predominantly O25:H4 (27.0%; *n* = 10) and O102:H6 (18.9%; *n* = 7) serotypes were detected. In addition, plasmid replicons were detected in 92.3% (*n* = 36) of the ESBL-EC, with the most common replicon types being IncFII (63.9%; *n* = 23), IncFIB (50.0%; *n* = 18), and IncFIA (38.9%; *n* = 14) (Table 2).

**Table 2:** Sequence types, serotypes, and plasmid replicons of sequenced ESBL-EC and ESBL-KP.

Variables			Frequency	Percentages
Isolates	Characteristics	Categories	*N*	%
ESBL-EC (*N* = 39)	STs	131	12	30.7
		648	11	28.2
		167	4	10.2
		617	2	5.1
		Others^a^	10	25.6
	Serotypable	Yes	37	94.9
		No	2	5.1
	Serotypes	O25:H4	10	27.0
		O102:H6	7	18.9
		0101:H21	4	10.8
		O153:H6	4	10.8
		Others^b^	10	27.0
	Detected plasmid replicon	Yes	36	92.3
		No	3	7.7
	Plasmid replicons	IncFII	23	63.9
		IncFIB	18	50.0
		IncFIA	14	38.9
		Col440I	6	16.7
		Others^c^	7	19.4
ESBL-KP (*N* = 49)	STs	2390	12	24.5
		17	9	18.4
		280	6	12.2
		14	5	10.2
		Others^d^	17	34.7
	Serotypable	Yes	45	91.8
		No	4	8.2
	Serotypes	O1ab:K16	12	26.7
		O1ab:K2	6	13.3
		O2afg:K23	6	13.3
		O5:K25	4	8.9
		Others^e^	13	28.9
	Detected plasmid replicon	Yes	49	100
		No	0	0.0
	Plasmid replicons	IncFII	41	83.7
		IncFIB	39	79.6
		IncR	18	36.7
		repB	14	28.6
		Others^f^	17	34.7

ESBL-EC.

ESBL-KP.

a Others: ST-10 (*n* = 1), ST-156 (*n* = 1), ST-224 (*n* = 1), ST-410 (*n* = 1), ST-1193 (*n* = 1), ST-1380 (*n* = 1), ST-2852 (*n* = 1), ST-3580 (*n* = 2), and ST-4981 (*n* = 1).

b Others: O101:H10 (*n* = 3), O16:H5 (*n* = 2), O101:H9 (*n* = 1), O8:H12 (*n* = 2), O17/O77:H18 (*n* = 1), O75:H5 (*n* = 1), O8:H23 (*n* = 1), and O8:H7 (*n* = 1).

c Others: IncQ1 (*n* = 2), IncX4 (*n* = 2), IncY (*n* = 3), IncI1-I (*n* = 3), and ColB (*n* = 1).

d Others: ST-20 (*n* = 1), ST-39 (*n* = 1), ST-45 (*n* = 3), ST-48 (*n* = 1), ST-54 (*n* = 2), ST-101 (*n* = 1), ST-307 (*n* = 3), ST-340 (*n* = 1), ST-607 (*n* = 1), ST-966 (*n* = 1), and ST-985 (*n* = 2).

e Others: O2afg:KL102 (*n* = 2), O2afg:KL122 (*n* = 2), O3:K20 (*n* = 2), O3b:K14 (*n* = 2), O1ab:K17 (*n* = 1), O1ab:K23 (*n* = 1), O1ab:K25 (*n* = 1), O1ab:K24 (*n* = 1), O1ab:K39 (*n* = 1), O1ab:K62 (*n* = 1), O2a:K24 (*n* = 1), O3b:K71 (*n* = 1), and O4:K15 (*n* = 1).

f Others: Col440II (*n* = 9), Col440I (*n* = 3), IncM1 (*n* = 2), IncHI1B (*n* = 1), p0111 (*n* = 1), and IncFIA (*n* = 1).

All ESBL-KP were identified by Kleborate as *K. pneumoniae* subsp. *pneumoniae*. Fifteen STs were detected, with ST2390 (24.5%; *n* = 12) and ST17 (18.4%; *n* = 9) being predominant. The ST2390 was reported in Tanzania for the first time in this study. Notably, 10 of the 12 ESBL-KP ST2390 isolates were recovered from blood samples. Most ESBL-KP (91.8%; *n* = 45) were serotypable, with frequent serotypes including O1ab:K16 (26.7%; *n* = 12), O1ab:K2 (13.3%; *n* = 6), and O2afg:K23 (13.3%; *n* = 6). Plasmid replicons were detected in all ESBL-KP isolates, with the most common replicons being IncFII (83.7%; *n* = 41), IncFIB (79.6%; *n* = 39), and IncR (36.7%; *n* = 18). Plasmid replicon repB was exclusively detected in ESBL-KP and notably in all ESBL-KP ST2390 (*n* = 12) (Table 2).

### 3.3 Phenotypic antimicrobial resistance patterns

**Fig. 1:**
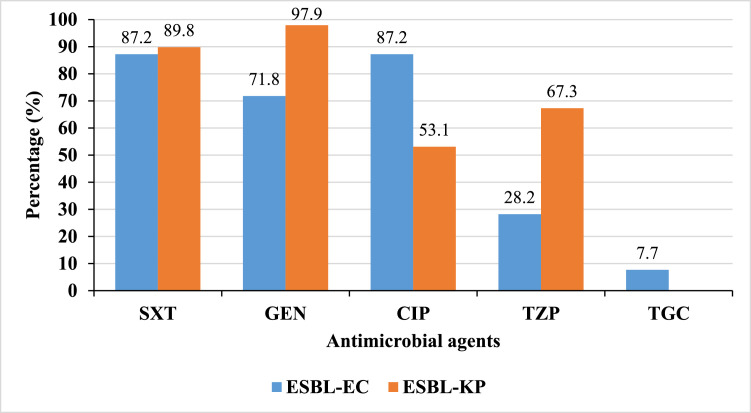
Percentage resistance of ESBL-EC (blue bars) and ESBL-KP (orange bars) against antimicrobial agents. Key: CIP = ciprofloxacin; GEN = gentamicin; TGC = tigecycline (ESBL-EC only); TZP = piperacillin-tazobactam; and SXT = trimethoprim-sulfamethoxazole.

ESBL-PE demonstrated high resistance rates (>50%) to most antimicrobial agents, with the exception of meropenem, against which no resistance was detected in either ESBL-EC or ESBL-KP. ESBL-EC exhibited significantly higher resistance to ciprofloxacin compared to ESBL-KP (87.2% vs. 53.1%, *P* < 0.01), whereas ESBL-KP showed markedly greater resistance to gentamicin (97.9% vs. 71.8%, *P* < 0.01) and piperacillin-tazobactam (67.3% vs. 28.2%, *P* < 0.01). Low resistance to tigecycline was observed among ESBL-EC (7.7%; 3/39); however, this agent was not tested for ESBL-KP, and thus, corresponding results are not presented (Fig. 1).

### 3.4 Genomic determinants of AMR in ESBL-EC and ESBL-KP

The *bla*_CTX−M-15_ gene was the predominant determinant of resistance to third-generation cephalosporins (3GCs), and thus ESBL production, in both ESBL-EC (87.2%; *n* = 34) and ESBL-KP (95.9%; *n* = 47). Despite this similarity, notable differences were observed in the distribution of other ARGs between the two species, ESBL-EC and ESBL-KP. For aminoglycoside resistance, *aac*(6′)-Ib-cr was most common in ESBL-EC (61.5%), whereas *aac*(3)-IIa predominated in ESBL-KP (67.3%). Regarding trimethoprim resistance, *dfr*A17 (61.5%) was most frequent in ESBL-EC, while *Oqx*B (53.1%) was predominant in ESBL-KP. Similarly, for amphenicol resistance, *cat*B3 (43.6%) was the leading gene in ESBL-EC, compared to *Oqx*B (53.1%) in ESBL-KP. Interestingly, *fos*A (36.7%; *n* = 18) and *arr*-3 (30.6%; *n* = 15), conferring resistance to fosfomycin and rifamycin, respectively, were detected exclusively in ESBL-KP (Table 3).

**Table 3:** Genomic determinants of AMR in ESBL-EC and ESBL-KP.

Antimicrobial class	Resistance gene	ESBL-EC (*N* = 39)	ESBL-KP (*N* = 49)
		*n* (%)	*n* (%)
Beta-lactams	*bla*_CTX−M-15_	34 (87.2)	47 (95.9)
	*bla*_TEM-1B_	26 (66.7)	39 (82.6)
	*bla*_OXA-1_	24 (61.5)	13 (26.5)
	*bla*_SHV-65_	–	12 (24.5)
	*bla*_SHV-1_	–	10 (20.4)
	*bla*_SHV-100_	–	7 (14.3)
	Others	5 (13.0)	20 (40.8)
Aminoglycosides	*aac*(6′)-Ib-cr	24 (61.5)	30 (61.2)
	*aad*A5	20 (51.3)	1 (2.0)
	*aph*(6)-Id	17 (43.6)	26 (53.1)
	*aac*(3)-IIa	15 (38.5)	33 (67.3)
	*aph*(3″)-Ib	9 (23.1)	11 (22.4)
	*aad*A2	3 (7.7)	1 (2.0)
	*aph*(3′)-Ia	2 (5.1)	12 (24.5)
	*aac*(3)-IId	2 (5.1)	6 (12.2)
Aminocyclitol	*aad*A5	20 (51.3)	1 (2.0)
	*aad*A2	3 (7.7)	1 (2.0)
	*aad*A16	–	10 (20.4)
	*aad*A1	–	1 (2.0)
Quinolones	*aac*(6′)-Ib-cr	24 (61.5)	30 (61.2)
	*qnr*S1	5 (12.8)	–
	*qep*A4	1 (2.6)	–
	*Oqx*B	–	26 (53.1)
	*qnr*B1	–	8 (16.3)
Sulfamethoxazole	*sul*1	23 (59.0)	14 (28.6)
	*sul*2	17 (43.6)	34 (69.4)
	*sul*3	1 (2.6)	–
Trimethoprim	*dfr*A17	24 (61.5)	–
	*dfr*A14	4 (10.3)	13 (26.5)
	*dfr*A12	3 (7.7)	–
	*dfr*A1	1 (2.6)	–
	*OqxB*	–	26 (53.1)
	*dfr*A27	–	3 (6.1)
	*dfr*A72	–	2 (4.1)
Tetracyclines	*tet*(A)	19 (48.7)	20 (40.8)
	*tet*(B)	15 (38.5)	–
	*tet*(D)	–	12 (24.5)
Amphenicols	*cat*B3	17 (43.6)	–
	*OqxB*	–	26 (53.1)
	*cat*A2	–	2 (4.1)
Fosfomycin	*fos*A	–	18 (36.7)
Rifamycin	*arr*-3	-	15 (30.6)

Others: ESBL-EC: *bla*_OXA-320_ (*n* = 1), *bla*_OXA-534_ (*n* = 1), *bla*_TEM-34_ (*n* = 1), *bla*_TEM-206_ (*n* = 1), and *bla*_TEM-216_ (*n* = 1) and ESBL-KP: *bla*_SHV-187_ (*n* = 3), *bla*_SHV-28_ (*n* = 2), *bla*_SHV-148_ (*n* = 2), *bla*_SHV-172_ (*n* = 2), *bla*_SHV-27_ (*n* = 1), *bla*_SHV-56_ (*n* = 1), *bla*_SHV-106_ (*n* = 1), *bla*_SHV-161_ (*n* = 1), *bla*_SHV-182_ (*n* = 1), *bla*_TEM-1C_ (*n* = 1), *bla*_TEM-30_ (*n* = 1), *bla*_TEM-234_ (*n* = 1), *bla*_OXA-320_ (*n* = 1), *bla*_OXA-534_ (*n* = 1), and *bla*_CTX−M-27_ (*n* = 1).

Five ESBL-EC lacked detectable 3GCs-resistance genes when screened using ResFinder at the CGE. However, further analysis with the RGI revealed the presence of genes encoding AmpC β-lactamases, including chromosomally encoded variants (EC-8, EC-15, and Ecol_ampC_BLA) and the plasmid-mediated gene *bla*_CMY-181_. Overall, discrepancies were noted between genotypic and phenotypic resistance profiles, underscoring the complexity of resistance mechanisms in these Yisolates (Supplementary Table 1).

### 3.5 Genomic determinants of virulence factors in ESBL-EC and ESBL-KP

In ESBL-EC, the detected virulence genes (VGs) were classified into seven functional categories: (i) adhesion and colonisation, (ii) capsule and biofilm formation, (iii) invasion and intracellular survival, (iv) immune evasion and serum resistance, (v) iron acquisition systems, (vi) toxin production, and (vii) antibacterial activity and survival. The most prevalent genes included *fim*H (89.7%), *asl*A (66.7%), and *csg*A (66.7%) encoding adhesion and colonization; *nlp*I and *yeh*A/B/C/D (100%, each) encoding capsule and biofilm formation; *iha* (38.5%) associated with invasion and intracellular survival; *ter*C (100%) and *tra*J/T (64.1%) encoding immune evasion and serum resistance; *fyu*A (82.1%), *irp*2 (82.1%), and *sit*A (76.9%) encoding iron acquisition systems; *hlyA*/E/F (74.4%) encoding pore forming haemolysins/toxins; and *cma* and *clp*K1 (5.1%, each) encoding proteins associated with antibacterial activity and survival (Table 4).

**Table 4:** Proportions and distributions of genes encoding virulence factors in ESBL-EC.

Functional categories	Virulence gene (VG)	Frequency	Percentages
		*n*	%
Adhesion and colonisation	*fim*H	35	89.7
	*asl*A	26	66.7
	*csg*A	26	66.7
	*yfc*V	25	64.1
	*Hra*	22	56.4
	*lpf*A	18	46.2
	*afa*A/B/C/D/E	16	41.0
	*pap*A/C	17	43.6
	*fde*C	4	10.3
	*fae*F	1	2.6
	*aap*	1	2.6
	*agg*R/4C	2	5.1
Capsule and biofilm formation	*nlp*I	39	100
	*yeh*A/B/C/D	39	100
	*kps*E/M	25	64.1
	*cap*U	1	2.6
Invasion and intracellular survival	*iha*	15	38.5
	*Tia*	2	5.1
	*Cia*	2	5.1
Immune evasion and serum resistance	*ter*C	39	100
	*tra*J/T	25	64.1
	*Iss*	18	46.2
	*omp*T	14	35.9
	*gad*	4	10.3
	*eat*A	1	2.6
	*usp*	1	2.6
Iron acquisition systems	*fyu*A	32	82.1
	*irp*2	32	82.1
	*sit*A	30	76.9
	*iut*A	27	69.2
	*chu*A	26	66.7
	*iuc*C	25	64.1
	*ire*A	8	20.5
	*mch*C/F	2	5.1
	*iro*N	1	2.6
	*neu*C	1	2.6
Toxins	*hly*A/E/F	29	74.4
	*sat*	13	33.3
	*cnf*1	11	28.2
	*sen*B	8	20.5
	*ast*A	4	10.3
	*sep*A	1	2.6
Antibacterial activity and survival	*cma*	2	5.1
	*clp*K1	2	5.1
	*col*E4	1	2.6

On the other hand, VGs identified in ESBL-KP were grouped into three main functional categories: (i) adhesion and colonisation, (ii) capsule formation and immune evasion, and (iii) iron acquisition systems. The genes *fim*A-I/K and *mrk*A-D/F/H/I/J associated with adhesion and colonisation; *acr*A/B and *rcs*A/B encoding capsule formation and immune evasion; and *ent*A-E/F/S, *fep*A-D/G, *fes*, and *iro*E associated with iron acquisition systems were detected in all ESBL-KP (Table 5). All ESBL-KP ST2390 isolates were found to harbour *ent*A-E/F/S, *fep*A-D/G, *fes*, and *iro*E, while *irp*1/2 and *ybt*A/E/P/Q/R/S/T/U/X were detected in 11 of the 12 isolates. According to the Kleborate virulence scoring system, 61.2% (30/49) of ESBL-KP isolates had a virulence score of 1.

**Table 5:** Proportions and distributions of genes encoding virulence factors in ESBL-KP.

Functional categories	Virulence gene (VG)	Frequency	Percentages
		*n*	%
Adhesion and colonisation	*fim*A/B/C/D/E/F/G/H/I/K	49	100
	*mrk*A/B/C/D/F/H/I/J	49	100
Capsule formation and immune evasion	*acr*A/B	49	100
	*rcs*A/B	49	100
	*wzi*	37	75.5
Iron acquisition systems	*ent*A/B/C/D/E/F/S	49	100
	*fep*A/B/C/D/G	49	100
	*Fes*	49	100
	*iro*E	49	100
	*irp*1/2	31	63.3
	*ybt*A/E/P/Q/R/S/T/U/X	30	61.2
	*fyu*A	30	61.2

### 3.6 Phylogenetic analysis of ESBL-PE

**Fig. 2:**
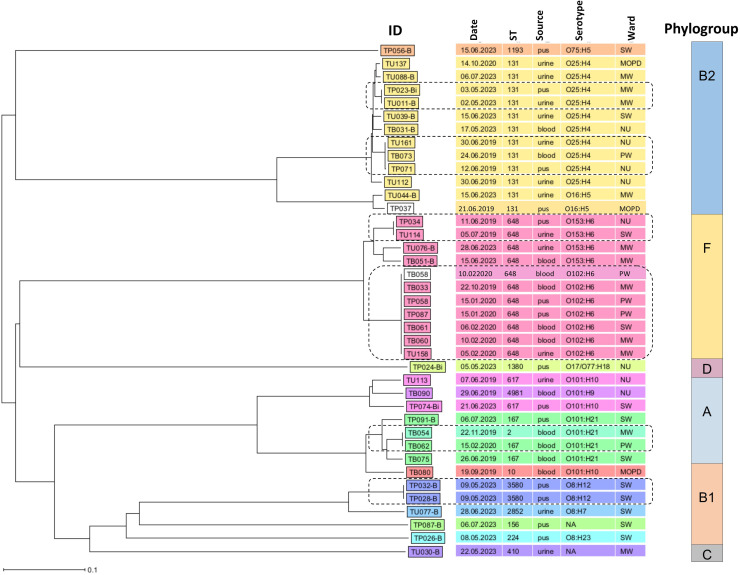
An NJ phylogenetic tree of ESBL-EC, based on cgMLST analysis. In the isolate IDs, the letter ‘T’ denotes Tanzania, followed by ‘B’, ‘U’, or ‘P’, which specify the sample type, blood, urine, and pus, respectively. The dashed rounded rectangles indicate clonal clusters corresponding to each phylogroup. Abbreviations: MOPD = medical outpatient; MW = medical ward; NU = neonatology unit; PW = paediatric ward; SW = surgical ward; NA = not serotypable.

The NJ phylogenetic tree based on cgMLST analysis of ESBL-EC isolates demonstrated considerable genetic diversity, identifying six phylogroups and clonal clusters within four phylogroups: B2, F, A, and B1. Phylogroup B2 had two clonal clusters of ST131, serotype O25:H4: the first clonal cluster with two strains from pus (TP23Bi; 3 May 2023; medical ward) and urine (TU11B; 2 May 2023; medical ward) and the second clonal cluster with three strains from urine (TU161; 30 June 2019; neonatology unit), blood (TB73; 24 June 2019; paediatric ward), and pus (TP71; 12 June 2019; neonatology unit). Phylogroup F had two clonal clusters: one clonal cluster contained pus (TP34; 11 June 2019; neonatology unit) and urine (TU114; 5 July 2019; surgical ward) isolates, both ST-648, serotype O153:H6, and the other clonal cluster comprised seven isolates from blood samples (TB33; 22 October 2019; medical ward), (TB58 and TB60: 10 February 2020; medical ward), (TB61; 6 February 2020; surgical ward), pus samples (TP58 and TP87: 1 January 2020; paediatric ward), and urine sample (TU158; 5 February 2020; medical ward), all ST-648, serotype O102:H6. Phylogroup A contained one clonal cluster of two blood isolates: TB54 (22 November 2019; medical ward) and TB62 (15 February 2020; paediatric ward) belonging to ST167, serotype O101:H21. Lastly, phylogroup B1 had one clonal cluster of two pus isolates: TP28B and TP32B (9 May 2023; surgical ward) of ST3580, serotype O8:H12 (Fig. 2).

**Fig. 3:**
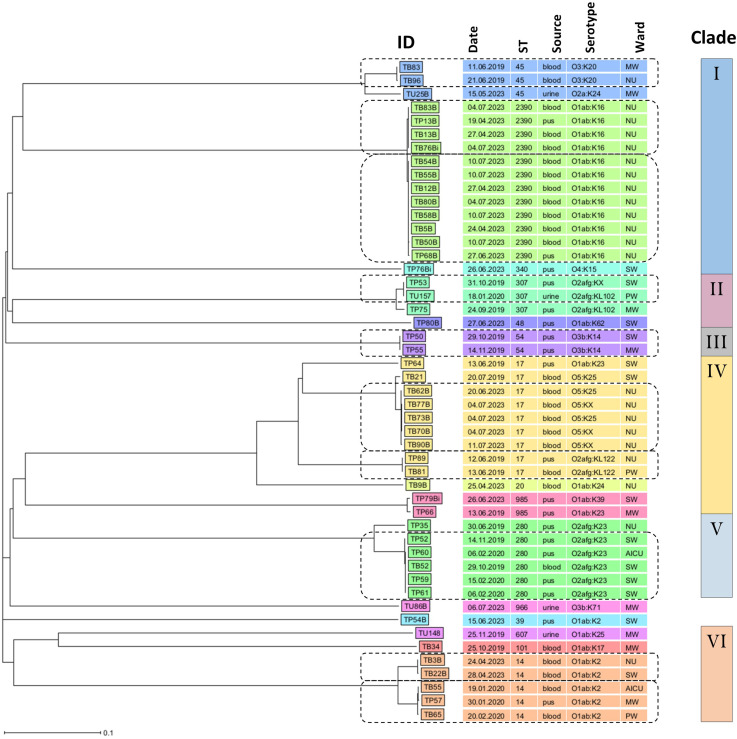
An NJ phylogenetic tree of ESBL-KP, based on cgMLST analysis. In the isolate IDs, the letter ‘T’ denotes Tanzania, followed by ‘B’, ‘U’, or ‘P’, which specify the sample type, blood, urine, and pus, respectively. The dashed rounded rectangles indicate clonal clusters corresponding to each clade. Abbreviations: AICU = adult intensive care unit; MW = medical ward; NU = neonatology unit; PW = paediatric ward; SW = surgical ward.

The cgMLST-based NJ phylogenetic analysis of ESBL-KP isolates revealed high genetic diversity, with six clades (as visually delineated by the authors) and clonal clusters within clades. Clade I comprised three clonal clusters: the first with two blood isolates (TB83; 11 June 2019; medical ward) and (TB96; 21 June 2019; neonatology unit) of ST45 and serotype O3:K20; the second with four isolates (ST2390, serotype O1ab: K16) from neonatology unit, including TB83B and TB76Bi (blood; 4 July 2023, both), TP13B (pus; 19 April 2023), and TB13B (blood; 27 April 2023); and the third with eight isolates (ST2390, serotype O1ab: K16) also from neonatology unit, including TB54B and TB55B (blood; 10 July 2023, both), TB12B (blood; 27 April 2023), TB80B (blood; 27 June 2023), TB58B (blood; 10 July 2023), TB5B (blood; 24 April 2023), TB50B (blood; 10 July 2023), and TP68B (pus; 27 June 2023). Clade II contained one clonal cluster of two isolates belonging to ST307: TP53 (pus; 31 October 2019; surgical ward) and TU157 (urine; 18 January 2020; paediatric ward). Clade III had once a clonal cluster of ST54, serotype O3b: K14, made of two isolates: TP50 (pus; 29 October 2019; surgical ward) and TP55 (pus; 14 November 2019; medical ward). Clade IV had two clonal clusters belonging to ST17: the first clonal cluster had five blood isolates, TB62B (20 June 2023), TB77B, TB73B, TB70B (4 July 2023, both) and TB90B (11 July 2023), all from neonatology unit, serotype O5:K25, and the second clonal cluster had two isolates, TP89 (pus; 12 June 2019; neonatology unit) and TB81 (blood; 13 June 2019; paediatric ward), serotype O2afg: KL122. Clade V had one clonal cluster of ST280, serotype O2afg: K23, made up of five isolates: TP52 (pus; 14 November 2019; surgical ward), TP60 (pus; 6 February 2020; AICU), TB52 (blood; 29 October 2019; surgical ward), TP59 (pus; 15 February 2020; surgical ward), and TP61 (pus; 6 February 2020; surgical ward). Clade VI contained two clonal clusters of ST14, serotype O1ab:K2: the first with two blood isolates TB3B (24 April 2023; neonatology unit) and TB22B (28 April 2023; surgical ward), and the second with three isolates, two from blood samples TB55 (19 January 2020; AICU) and TB65 (20 February 2020; paediatric ward), and one from pus sample TP57 (3 January 2020; medical ward) (Fig. 3).

## 4 Discussion

This comprehensive genomic epidemiological study reveals the presence of the global high-risk clone ESBL-EC ST131 and the emergence of ESBL-KP ST2390 reported in Tanzania for the first time in this study. The ESBL-KP ST2390 isolates harboured key virulence factors, notably *iro*E/N and *ybt*A/E/P/Q/R/S/T/U/X, suggesting that this ST may represent a potentially high-risk clone [27,28]. However, ESBL-KP ST2390 strains have been previously reported elsewhere, for instance, in China in 2019 [29]. Furthermore, we observed clonal transmission/expansion events of these global high-risk strains at this setting, mostly within neonatology units, highlighting potential gaps in infection prevention and control strategies.

In line with a previous study from the same setting [6] and elsewhere [30], we observed the predominance of ST131 phylogroup B2 in ESBL-EC. Alongside ST131 phylogroup B2, we identified ST648 phylogroup F as the second most prevalent lineage. Both ST131 phylogroup B2 and ST648 phylogroup F are well-documented extraintestinal pathogenic *E. coli* (ExPEC) lineages, characterised by their heightened virulence and extensive multidrug resistance [5,31]. Additionally, the dominance of uropathogenic ESBL-EC ST131 may be associated with specific virulence factors of this clone, including P fimbriae and type 1 fimbriae for superior bladder epithelial adhesion; iron-acquisition systems crucial for the iron-limited urinary tract environment; and increased biofilm-forming capacity on urinary bladder epithelium [32]. On the other hand, the predominance of ST2390 in ESBL-KP and particularly its clonal expansion in neonatology units, underscores the BSIs outbreak in the neonatology unit and the potential risk to vulnerable neonates. Generally, the predominance of *K. pneumoniae* in BSIs is favoured by its multiple and effective iron acquisition systems [33]. In contrast to the sustained dominance of ESBL-EC ST131, the predominant STs of ESBL-KP in our setting have fluctuated over time. For instance, Mshana *et al.* [7] identified ST14 as the leading clone in 2009–2010 [8], while Marando *et al.* later reported ST45 as predominant in 2016. The fluctuation of ESBL STs over time in our setting may partly be attributed to differences in the sampled patient populations or ward distributions. Additionally, despite the fluctuations of STs among ESBL-KP over time, the overall trend highlights the continued presence of a globally recognised high-risk clone within healthcare environments [34].

Consistent with previous studies [7,35], IncFIB was the most prevalent plasmid replicon among ESBL-EC and ESBL-KP, supporting a major role for conjugative IncF-type plasmids in the dissemination of resistance and virulence determinants [36]. Nevertheless, this study did not examine the specific localisation of ARGs or VFs within these plasmid replicons. Further, our observation of the dominance of high-risk global clone ESBL-EC ST131 and the newly detected ESBL-KP ST2390 highlights that clonal expansion contributes substantially to the spread of ARGs and rapid dissemination of AMR in our setting.

Notably, all ESBL-PE isolates in this study were susceptible to meropenem, aligning with previous studies in Tanzania [37] and Sri Lanka [38], underscoring its continued reliability as a last-resort agent for MDR infections, including those caused by ESBL-EC and ESBL-KP. This observation further illustrates the positive impact of antimicrobial stewardship efforts in curbing inappropriate use of this critical agent within our setting. However, consistent with previous studies in India [39] and Turkey [40], resistance to ciprofloxacin was significantly higher among ESBL-EC than ESBL-KP. Although ciprofloxacin resistance in ESBL-EC, particularly the ST131 clone, is predominantly mediated by chromosomal mutations [41], our findings show that most ESBL-EC isolates harboured acquired ciprofloxacin resistance genes. Conversely, we observed higher resistance to gentamicin and piperacillin-tazobactam among ESBL-KP than ESBL-EC. Collectively, these findings point to a species-specific dissemination of ARGs, likely driven by clonal expansion, as exemplified by the predominance of ESBL-EC ST131 and ESBL-KP ST2390 in our setting.

We observed the predominance of *bla*_CTX−M-15_ in both ESBL-EC and ESBL-KP, underscoring its widespread dissemination as a key determinant of 3GCs resistance among Enterobacterales. Nonetheless, we observed genotype-phenotype discordance, exemplified by the absence of detectable acquired resistance genes, particularly *bla*_CTX−M_ genes, in five ESBL-EC isolates. The AmpC β-lactamase-mediated resistance, an intrinsic mechanism in most *E. coli* [42], conferring resistance to β-lactams, including third-generation cephalosporins, may plausibly explain the ESBL-like phenotypes observed in the five ESBL-EC isolates in which no ESBL genes were detected. On the other hand, cryptic resistance genes present in the bacterial genome may remain phenotypically silent due to low expression levels, regulatory mutations, or suboptimal promoter activity, thereby accounting for the susceptible phenotypes observed among genotypically resistant isolates [43].

The virulence landscapes of ESBL-EC and ESBL-KP differ markedly in both diversity and functional emphasis. ESBL-EC harboured a wide array of VFs spanning multiple categories, indicative of high adaptability and opportunistic pathogenicity typical of ExPEC, such as ST131 [44]. By contrast, ESBL-KP exhibited a more conserved virulence profile, largely centred on adhesion, capsule production, and iron acquisition, underscoring the importance of colonisation and nutrient uptake. Additionally, the predominance of low Kleborate scores among ESBL-KP further suggests that multidrug resistance, rather than enhanced virulence, constitutes its primary advantage in healthcare-associated infections [45]. Nonetheless, a comprehensive understanding of VFs would require direct comparisons between 3GC-resistant and susceptible or ESBL-PE and non-ESBL-PE isolates, which were beyond the scope of this study.

The NJ phylogenetic analysis based on cgMLST revealed clonal clusters, suggesting potential intrahospital transmission of ESBL-PE. Specifically, this included the high-risk clone ESBL-EC ST131 and the newly identified ESBL-KP ST2390, occurring across patients and clinical conditions, particularly BSIs, UTIs, and SSTIs. Notably, ESBL-EC ST131 was detected circulating in medical, neonatology, and paediatric wards, while ST648 circulated within medical, surgical, and paediatric wards. In contrast, ESBL-KP ST2390 showed persistent circulation within the neonatology unit, indicative of extensive clonal expansion in this setting. Collectively, these findings point to shortcomings in IPC measures and suggest the potential role of environmental reservoirs or healthcare-associated transmission routes in sustaining these clones [46].

Importantly, our study aligns with the implementation gaps highlighted in the WHO-JEE 2023 assessment of Tanzania, which noted the country’s limited capacity for systematic genomic surveillance of AMR, IPC, and AMS [47]. By identifying an emerging ESBL-KP ST2390 in the neonatology unit and documenting its spread in critical care settings, our findings contribute to bridging this gap. However, significant challenges remain regarding nationwide representativeness, integration of genomic data into routine AMR surveillance, and linkage with clinical outcomes. Overall, the predominance of high-risk clone ST131 and the emergence of ST2390 underscore the urgent need to strengthen genomic surveillance systems and implement targeted infection prevention measures, particularly in vulnerable populations such as neonates, through improved hand hygiene, rigorous environmental monitoring, and strict contact precautions.

### 4.1 Study limitations

This single-centre study may limit generalizability to other healthcare settings in Tanzania or similar LMICs. The relatively small sample size and lack of environmental screening may have underestimated transmission pathways. Additionally, the absence of patient outcome data prevents assessment of the clinical impacts of these genomic changes.

## 5 Conclusion

This study provides a comprehensive WGS-based genomic epidemiological characterisation of ESBL-EC and ESBL-KP causing BSIs, UTIs, and SSTIs at Bugando Medical Centre in Mwanza, Tanzania. The presence of globally recognised high-risk clone ESBL-EC ST131 and the emerging ESBL-KP ST2390, together with evidence of clonal clusters, especially within medical wards and neonatology units, highlights substantial gaps in IPC. The dominance of *bla*_CTX−M-15_, species-specific resistance patterns, and distinct virulence landscapes underscore the role of clonal expansion in driving AMR. Collectively, these findings emphasise the urgent need to strengthen genomic surveillance, integrate WGS into routine AMR monitoring, and implement targeted IPC strategies to mitigate the spread of high-risk ESBL-producing pathogens in resource-limited healthcare settings.

## Appendix Supplementary materials

Image, application 1

## References

[1] Abay G.K., Shfare M.T., Teklu T.G., Kidane K.M., Gebremeskel T.K., Kahsay A.G. (2025). Extended-spectrum β-lactamase production and antimicrobial resistance among Enterobacteriaceae causing clinical infections in Africa: a systematic review and meta-analysis (2012–2020). Eur J Med Res.

[2] Murray C.J., Ikuta K.S., Sharara F., Swetschinski L., Aguilar G.R., Gray A. (2022). Global burden of bacterial antimicrobial resistance in 2019: a systematic analysis. The Lancet.

[3] Pokharel S., Raut S., Adhikari B. (2019). Tackling antimicrobial resistance in low-income and middle-income countries. BMJ Specialist J.

[4] Mazumder R., Hussain A., Abdullah A., Islam M.N., Sadique M.T., Muniruzzaman S. (2021). International high-risk clones among extended-spectrum β-lactamase–producing *Escherichia coli* in Dhaka, Bangladesh. Front Microbiol.

[5] Peirano G., Chen L., Kreiswirth B.N., Pitout J.D. (2020). Emerging antimicrobial-resistant high-risk *Klebsiella pneumoniae* clones ST307 and ST147. Antimicrob Agents Chemother..

[6] Seni J., Peirano G., Mshana S.E., Pitout J.D., DeVinney R. (2021). The importance of Escherichia coli clonal complex 10 and ST131 among Tanzanian patients on antimicrobial resistance surveillance programs. Eur JClin Microbiol Infect Dis.

[7] Mshana S.E., Hain T., Domann E., Lyamuya E.F., Chakraborty T., Imirzalioglu C. (2013). Predominance of Klebsiella pneumoniae ST14 carrying CTX-M-15 causing neonatal sepsis in Tanzania. BMC Infect Dis.

[8] Marando R., Seni J., Mirambo M.M., Falgenhauer L., Moremi N., Mushi M.F. (2018). Predictors of the extended-spectrum-beta-lactamases producing Enterobacteriaceae neonatal sepsis at a tertiary hospital, Tanzania. Int J Med Microbiol.

[9] WHO (2016).

[10] WHO publishes list of bacteria for which new antibiotics are urgently needed. (2017). [https://www.who.int/news/item/27-02-2017-who-publishes-list-of-bacteria-for-which-new-antibiotics-are-urgently-needed]. Accessed: June 1, 2022.

[11] WHO: WHO bacterial priority pathogens list, 2024: bacterial pathogens of public health importance to guide research, development and strategies to prevent and control antimicrobial resistance. Geneva, Switzerland; World Health Organization; 2024.

[12] URT: The national action plan on antimicrobial resistance 2017 - 2022, UnitedRepublic of Tanzania. Ministry of Health (URT); 2017.

[13] Silago V., Oravcova K., Matthews L., Mshana S.E., Claus H., Seni J. (2024). Epidemiology and antimicrobial resistance trends of pathogens causing urinary tract infections in Mwanza, Tanzania: a comparative study during and after the implementation of the National Action Plan on Antimicrobial Resistance (2017-2022). Int J Infect Dis.

[14] Silago V., Matthews L., Oravcova K., Mshana S.E., Seni J., Claus H. (2025). Epidemiology and antimicrobial resistance trends of bloodstream infections during and after the implementation of the National Action Plan on Antimicrobial Resistance in Mwanza, Tanzania: a comparative cross-sectional study. Infect Drug Resist.

[15] Procop G.W., Church D.L., Hall G.S., Janda W.M. (2020).

[16] Hudzicki J. (2009). Kirby-Bauer disk diffusion susceptibility test protocol. Am Soc Microbiol.

[17] EUCAST (2023).

[18] Joensen K.G., Tetzschner A.M., Iguchi A., Aarestrup F.M., Scheutz F. (2015). Rapid and easy in silico serotyping of *Escherichia coli* isolates by use of whole-genome sequencing data. J Clin Microbiol..

[19] Camacho C., Coulouris G., Avagyan V., Ma N., Papadopoulos J., Bealer K. (2009). BLAST+: architecture and applications. BMC Bioinformat.

[20] Carattoli A., Zankari E., Garcìa-Fernandez A., Larsen M.V., Lund O., Villa L. (2014). PlasmidFinder and pMLST: in silico detection and typing of plasmids. Antimicrob Agents Chemother.

[21] Bortolaia V., Kaas R.S., Ruppe E., Roberts M.C., Schwarz S., Cattoir V. (2020). Florensa AF: resFinder 4.0 for predictions of phenotypes from genotypes. J Antimicrob Chemother.

[22] Alcock B.P., Huynh W., Chalil R., Smith K.W., Raphenya A.R., Wlodarski M.A. (2023). CARD 2023: expanded curation, support for machine learning, and resistome prediction at the Comprehensive Antibiotic Resistance Database. Nucleic Acids Res..

[23] Lam M.M., Wick R.R., Watts S.C., Cerdeira L.T., Wyres K.L., Holt K.E. (2021). A genomic surveillance framework and genotyping tool for *Klebsiella pneumoniae* and its related species complex. Nat Commun.

[24] Beghain J., Bridier-Nahmias A., Le Nagard H., Denamur E., Clermont O. (2018). ClermonTyping: an easy-to-use and accurate in silico method for Escherichia genus strain phylotyping. Microb Genom.

[25] Clermont O., Dixit O.V., Vangchhia B., Condamine B., Dion S., Bridier-Nahmias A. (2019). Characterization and rapid identification of phylogroup G in *Escherichia coli*, a lineage with high virulence and antibiotic resistance potential. Environ Microbiol..

[26] StataCorp (2017).

[27] Chen J., Zhang H., Liao X. (2023). Hypervirulent *Klebsiella pneumoniae*. Infect Drug Resist.

[28] Tang Y., Du P., Du C., Yang P., Shen N., Russo T.A. (2025). Genomically defined hypervirulent *Klebsiella pneumoniae* contributed to early-onset increased mortality. Nat Commun.

[29] Meng X., Yang J., Duan J., Liu S., Huang X., Wen X. (2019). Assessing molecular epidemiology of carbapenem-resistant *Klebsiella pneumoniae* (CR-KP) with MLST and MALDI-TOF in Central China. Sci Rep.

[30] Muller A., Gbaguidi-Haore H., Cholley P., Hocquet D., Sauget M., Bertrand X. (2021). Hospital-diagnosed infections with *Escherichia coli* clonal group ST131 are mostly acquired in the community. Sci Rep.

[31] Schaufler K., Semmler T., Wieler L.H., Trott D.J., Pitout J., Peirano G. (2019). Genomic and functional analysis of emerging virulent and multidrug-resistant *Escherichia coli* lineage sequence type 648. Antimicrob Agents Chemother..

[32] Nicolas-Chanoine M-H, Bertrand X., Madec J-Y (2014). Escherichia coli ST131, an intriguing clonal group. Clin Microbiol Rev.

[33] Lan P., Lu Y., Fu Y., Yu Y., Zhou J. (2025). Siderophores and beyond: a comprehensive review of iron acquisition in *Klebsiella pneumoniae*. Virulence.

[34] Peirano G., Pitout J.D. (2019). Extended-spectrum β-lactamase-producing Enterobacteriaceae: update on molecular epidemiology and treatment options. Drugs.

[35] Byarugaba D.K., Osman T.S., Sayyouh O.M., Wokorach G., Kigen C.K., Muturi J.W. (2024). Genomic epidemiology of multidrug-resistant *Escherichia coli* and *Klebsiella pneumoniae* in Kenya, Uganda, and Jordan. Emerging Infect Dis.

[36] Rafaï C., Frank T., Manirakiza A., Gaudeuille A., Mbecko J-R, Nghario L. (2015). Dissemination of IncF-type plasmids in multiresistant CTX-M-15-producing Enterobacteriaceae isolates from surgical-site infections in Bangui, Central African Republic. BMC Microbiol..

[37] Mshana H.J., Kovacs D., Muro F., Zadoks R., Oravcova K., Matthews L. (2025). The burden, risk factors, and antimicrobial susceptibility pattern associated with extended-spectrum beta-lactamase-producing *E. coli* and *K. pneumoniae* carriage among neonates and their surroundings at a referral hospital in the Moshi municipality. Front. Antibiot.

[38] Fernando M., Luke W., Miththinda J., Wickramasinghe R., Sebastiampillai B., Gunathilake M. (2017). Extended spectrum beta-lactamase producing organisms causing urinary tract infections in Sri Lanka and their antibiotic susceptibility pattern–a hospital based cross sectional study. BMC Infect. Dis..

[39] Mandal J., Acharya N.S., Buddhapriya D., Parija S.C. (2012). Antibiotic resistance pattern among common bacterial uropathogens with a special reference to ciprofloxacin-resistant *Escherichia coli*. Ind J Med Res.

[40] Tolun V., Küçükbasmaci Ö, Törümküney-Akbulut D., Catal C., Anğ-Küçüker M., Auğ Ö (2004). Relationship between ciprofloxacin resistance and extended-spectrum β-lactamase production in Escherichia coli and *Klebsiella pneumoniae* strains. Clin Microbiol Infect.

[41] Amel R., Abderrazek B., Sana F., Ahmed S., Mariem Z., Lamia K. (2024). Molecular mechanisms impact on fluoroquinolone resistance among *E. coli* from enteric carriage monitoring before prostate biopsy and earliest description of qnr B81. Sci Rep.

[42] Bajaj P., Singh N.S., Virdi J.S. (2016). *Escherichia coli* β-lactamases: what really matters. Front Microbiol.

[43] Deekshit V.K., Srikumar S. (2022). To be, or not to be’—the dilemma of ‘silent’ antimicrobial resistance genes in bacteria. J Appl Microbiol.

[44] Sora V.M., Meroni G., Martino P.A., Soggiu A., Bonizzi L., Zecconi A. (2021). Extraintestinal pathogenic *Escherichia coli*: virulence factors and antibiotic resistance. Pathogens.

[45] Pristas I., Ujevic J., Bodulić K., Andrijasevic N., Bedenic B., Payerl-Pal M. (2024). The association between resistance and virulence of *Klebsiella pneumoniae* in high-risk clonal lineages ST86 and ST101. Microorganisms.

[46] Adler A., Gniadkowski M., Baraniak A., Izdebski R., Fiett J., Hryniewicz W. (2012). Transmission dynamics of ESBL-producing *Escherichia coli* clones in rehabilitation wards at a tertiary care centre. Clin Microbiol Infect.

[47] World Health Organization (2022).

